# Evaluating area-level spatial clustering of *Salmonella* Enteritidis infections and their socioeconomic determinants in the greater Toronto area, Ontario, Canada (2007 – 2009): a retrospective population-based ecological study

**DOI:** 10.1186/1471-2458-13-1078

**Published:** 2013-11-15

**Authors:** Csaba Varga, David L Pearl, Scott A McEwen, Jan M Sargeant, Frank Pollari, Michele T Guerin

**Affiliations:** 1 Department of Population Medicine, Ontario Veterinary College, University of Guelph, Guelph, ON N1G 2 W1, Canada; 2 Ontario Ministry of Agriculture, Food and Rural Affairs, Guelph, ON N1G 4Y2, Canada; 3 Centre for Public Health and Zoonoses, Ontario Veterinary College, University of Guelph, Guelph, ON N1G 2 W1, Canada; 4 Centre for Foodborne, Environmental and Zoonotic Infectious Diseases, Public Health Agency of Canada, Guelph, ON N1H 8 J1, Canada

**Keywords:** *Salmonella* Enteritidis, Socioeconomic status, Spatial scan statistic, GIS, Choropleth map, Negative binomial regression, Moran’s I, Ecological study

## Abstract

**Background:**

There have been only a few region-level ecological studies conducted in Canada investigating enteric infections in humans. Our study objectives were to 1) assess the spatial clustering of *Salmonella enterica* serotype Enteritidis (*S.* Enteritidis) human infections in the Greater Toronto Area, and 2) identify underlying area-level associations between *S.* Enteritidis infection rates and socioeconomic status (SES) indicators that might explain the clustering of infections.

**Methods:**

Retrospective data on *S.* Enteritidis infections from 2007 to 2009 were obtained from Ontario’s reportable disease surveillance database and were grouped at the forward sortation area (FSA) - level. A spatial scan statistic was employed to identify FSA-level spatial clusters of high infection rates. Negative binomial regression was used to identify FSA-level associations between *S.* Enteritidis infection rates and SES indicators obtained from the 2006 Census of Canada. Global Moran’s I statistic was used to evaluate the final model for residual spatial clustering.

**Results:**

A spatial cluster that included nine neighbouring FSAs was identified in downtown Toronto. A significant positive curvilinear relationship was observed between *S*. Enteritidis infection rates and FSA-level average number of children at home per census family. Areas with high and areas with low average median family income had higher infection rates than FSAs with medium average median family income. Areas with a high proportion of visible minority population had lower infection rates than FSAs with a medium proportion of visible minority population. The Moran’s I statistic was not significant, indicating that no residual spatial autocorrelation was present after accounting for the SES variables in the final model.

**Conclusions:**

Our study demonstrated that FSAs with high and low average median family income, medium proportion of visible minority population, and high average number of children at home per census family had the highest *S*. Enteritidis infection rates. These areas should be targeted when designing disease control and prevention programs. Future studies are needed in areas with high *S*. Enteritidis infection rates to identify sources of environmental contamination of the local food supply, to assess food safety practices at local food markets, retail stores, and restaurants, and to identify novel individual-level risk factors.

## Background

Non-typhoidal salmonellosis (NTS) is a major foodborne zoonotic infection that poses a significant public health risk [1-5]; on a global basis, it affects an estimated 93.8 million people and causes 155,000 deaths annually [1]. In Canada, NTS is the second most frequently reported enteric infection [6], and results in the largest number of hospitalizations and deaths among foodborne diseases [7]. Surveillance systems in Canada have identified an increasing trend of *Salmonella enterica* serovar Enteritidis (*S.* Enteritidis) infections in humans, with a threefold increase between 2003 and 2009 [8]. As a result of this increase, *S.* Enteritidis became the most common *Salmonella* serotype in Ontario and Canada.

There are an abundance of case–control studies worldwide that have evaluated individual-level associations between *S.* Enteritidis infections and potential risk factors including: consumption of chicken [9,10], consumption of raw or undercooked eggs [9,11,12], person-to-person transmission through infected food handlers [13-15], animal-to-person transmission [16,17], and international travel [9,18-20]. In contrast, there are a limited number of population-based ecological studies that have evaluated area-level associations between enteric infections and socioeconomic status (SES) indicators. Ecological and individual-level studies from Canada and Europe [21-24] have demonstrated associations between enteric infections and SES determinants (e.g. household income, education level, unemployment rate, number of children per household, cultural group, population density). Two ecological studies from the United States of America (US) analyzed associations between salmonellosis and area-level SES and sociodemographic factors. Younus et al. [25] found that areas with higher education attainment had a greater incidence of salmonellosis compared to areas with a lower education attainment, which was partly explained by the overrepresentation of highly educated persons in the surveillance system due to their better access to health care and their willingness to visit a doctor even for mild symptoms. It has also been hypothesized that groups with higher education tend to eat outside of the home more frequently and are more likely to own pets [25], which are considered to be sources of *Salmonella* for individuals [26,27]. In another US ecological study, Chang et al. [28] showed that areas with a higher black, Hispanic, or Latino population had a higher likelihood of *Salmonella* infections than areas with a predominantly white population. However, *S.* Enteritidis infections might have different SES risk factors than other *Salmonella* serotypes.

**Figure 1:**
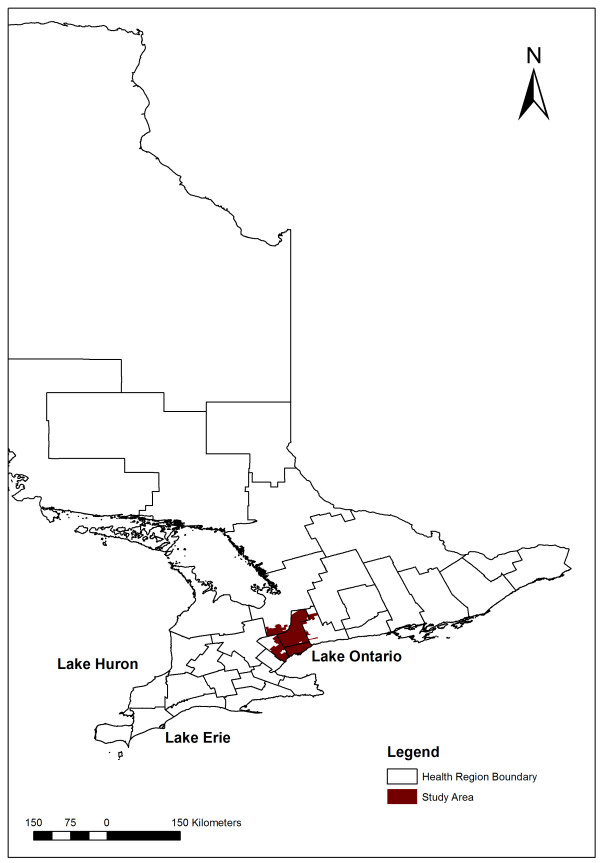
Location of the study area within the Greater Toronto Area in Ontario, Canada.

We previously examined the incidence, seasonality, and demographic risk factors of *S.* Enteritidis human infections across Ontario’s health regions from 2007 to 2009 [29] and identified three public health units (PHUs) within the Greater Toronto Area (GTA) with moderate to high incidence rates that were of interest for further assessment (Figure 1). Therefore, our objectives were to assess forward sortation area (FSA; first three digits of the postal code)-level clustering of *S.* Enteritidis infections within these three PHUs, and identify underlying area-level associations between standardized incidence rates (SIRs) of *S.* Enteritidis infections and SES risk factors. The findings of this study are expected to assist public health authorities in designing effective area-based prevention and control programs for *S.* Enteritidis.

## Methods

### Study population and study design

In Ontario, there are 36 PHUs mandated by the provincial ministry of health to manage health promotion and disease prevention programs. Our study area included three PHUs within the GTA, namely, the City of Toronto Health Unit, Peel Regional Health Unit, and York Regional Health Unit. The analysis was conducted at the FSA**-**level, a well-defined zone within a larger geographic region, represented by the first three characters of the postal code. We excluded FSAs with less than 500 residents (n = 12) to obtain stable incidence rates, and FSAs with centroids outside of the three PHU boundaries (n = 7) to attain a well-delimited and uniform geographical study area. We included 153 FSAs containing a combined population of 4,570,151 that accounted for 40% of Ontario’s population in 2006 (Figure 1). The annual population sizes of the 153 FSAs ranged from 2,172 to 84,180 persons, with a mean of 29,870 persons.

### Data sources

#### i) Ontario Ministry of Health and Long-Term Care, integrated Public Health Information System

In Ontario, a confirmed case of salmonellosis is defined as the isolation of *Salmonella* spp. (excluding *Salmonella* Typhi or Paratyphi) from an appropriate clinical sample (e.g. stool, urine, blood) with or without clinically-compatible signs and symptoms [30]. Salmonellosis is a reportable disease under provincial legislation [31]; all infections confirmed by hospital, private, and public health laboratories must be reported to the local PHU, whose personnel are required to investigate each case and enter the case’s demographic information (age, sex, area of residence), clinical features, and exposure history to the MOHLTC through iPHIS. In addition, all clinical *Salmonella* isolates are sent to Toronto Public Health Laboratories for confirmation and serotyping using conventional methods [32]. Information pertaining to *S.* Enteritidis cases’ PHU, FSA, and date of onset of illness were extracted from iPHIS. Within the iPHIS system, individuals who experience a recurrent episode of a reportable disease receive the same case number. Therefore, we were able to use unique de-identified case numbers to identify recurrent episodes of *S.* Enteritidis infections. If duplicate case numbers were identified, only the first episode was included in the analysis. Our study data represent all *S.* Enteritidis infections from the three PHUs captured within the database between January 1, 2007 and December 31, 2009. During the study period, no major outbreaks were reported in the GTA. Reports obtained from iPHIS are useful for evaluating food source attribution and demographic risk factors, yet they lack SES indicators, such as family income, family size, or level of education, due to privacy legislation. Our data did not have any personal or health information that could be linked back to the original identifiers; therefore, the University of Guelph Ethics Review Board did not require ethics approval for this study.

#### ii) Census of Canada

The Census of Canada is administered every five years by Statistics Canada and collects self-reported demographic and socioeconomic characteristics on Canadians [33]. Census data are available for various geographic areas, including country, provinces and territories, health regions, and FSAs. Forward sortation area was selected as the unit of interest for our study because FSAs in the GTA have well-defined geographical boundaries; FSA-level SES indicators for the three PHUs of interest within the GTA were obtained from the 2006 Census.

### Statistical analysis

#### Descriptive statistics

The unadjusted *S.* Enteritidis incidence per 100,000 person-years per FSA over the study period was calculated by dividing the sum of the annual number of *S.* Enteritidis cases per FSA by the sum of the annual FSA population estimates obtained from the 2006 Census. A choropleth map of the unadjusted incidence was created using ArcGIS 10 (ESRI Inc., Redlands, CA, US); Jenk’s optimization classification method was used to define the critical intervals for mapping [34]. This method arranges data into classes based on their distribution by using an algorithm that reduces variance within groups and maximizes variance between groups.

In addition, the relative risk per FSA was calculated by dividing the observed number of cases per FSA by the expected number of cases per FSA (calculation described in the ‘Dependent variable’ subsection), and then mapped using ArcGIS 10 to illustrate FSAs with excess risk after adjusting for the age and sex distribution of the population.

### Spatial analysis

Date of illness onset was used to assign each case to a particular month and year. Each case was categorized into one of five age groups: 0–9, 10–24, 25–34, 35–49, and 50+ years. Children 0–9 were grouped into one category to account for children’s higher risk of enteric infections [16,21]. Adolescents and young adults were grouped together because of their similar rate of *S.* Enteritidis infections [29]. Adults 50 years of age and older were pooled into one category because of the small number of cases in this age group. Cartesian coordinates of latitude and longitude for each FSA centroid were calculated in ArcGIS 10. A database was created in a Microsoft Excel 2010 (Microsoft Corporation, Redmond, WA, US) spreadsheet containing each case identifier and its corresponding information (FSA, FSA population estimate, Cartesian coordinates of the FSA centroid, illness onset month and year, age, and sex), which was subsequently imported into the SaTScan™ software [35] for analysis.

To identify clusters with high infection rates we employed the retrospective, purely spatial scan statistic that used the discrete Poisson model [36]. Age and sex were added to the model to account for possible confounding effects. The model assumes that the number of cases in each FSA are Poisson-distributed, based on a known underlying population at risk. The scan statistic uses a circular scanning window of variable radii. The null hypothesis assumes equal infection rates inside the scanning window compared to outside the scanning window, whereas the alternative hypothesis expects a higher infection rate inside the scanning window compared to outside [37]. The scan statistic uses a maximum likelihood function that identifies high rate clusters that are least likely to have happened by chance alone. A p-value was obtained through Monte Carlo hypothesis testing [38] using 999 replications and a relative risk is allocated to this cluster. For our analysis, the maximum spatial circular window size was set to include up to 50% of the population at risk [37]. Secondary clusters were also reported if they did not overlap with the primary cluster. Spatial clusters significant at α = 0.05 were illustrated in a map using ArcGIS 10.

### Regression analysis

A regression analysis (described below) was employed to identify area-level associations between SIRs of *S.* Enteritidis infections and SES variables in order to better understand the underlying socioeconomic factors that might contribute to spatial clustering of *S.* Enteritidis infections within the three PHUs.

### Variable selection and creation

#### i) SES variables

According to the Census of Canada, a *census family* is a married couple (with or without children of either or both spouse), common-law couple (with or without children of either or both partner), or lone-parent family regardless of sex of parent. A couple may be of opposite or same sex. All members of a specific census family must live in the same household.

The following FSA-level SES indicators were obtained from the 2006 Census of Canada [33] and calculated as follows:

1) Average number of children at home per census family, which was calculated by summing the number of children per family then dividing by the total number of families.

2) Average number of persons per census family, which was calculated by summing the number of persons per family then dividing by the total number of families.

3) Average number of rooms per home, which was calculated by summing the number of rooms in a home then dividing by the total number of homes.

4) Immigrant population proportion, which was calculated by dividing the immigrant population by the total population and multiplying by 100. The term *immigrant* refers to a person who is, or has ever been, a landed immigrant in Canada. A *landed immigrant* is a person born outside of Canada who has been granted the right to live in Canada permanently by immigration authorities.

5) Average median family income, which was calculated by dividing the sum of the median income of families in a household by the total number of households. The Census dictionary defines the *median income of families in a household* as “that amount which divides family member’s income size distribution, ranked by size of income, into two halves. Median incomes of families in a household are normally calculated for all members of the family, whether or not they reported income”. The total income of the family members was defined as the sum of the income obtained from wages, salaries, bonuses, interest, and any provincial - federal benefits and welfare payments.

6) Proportion of university graduates of persons between 25 and 64 years of age, which was calculated by dividing the number of persons between 25 and 64 years of age with a university degree by the total population between 25 and 64 years of age and multiplying by 100.

7) Unemployment rate of persons 15 years of age or older, which was calculated by dividing the number of unemployed by the number of people in the labour force and multiplying by 100.

8) Visible minority population proportion, which was calculated by dividing the visible minority population by the total population and multiplying by 100. *Visible minority*, according to the Employment Equity Act, refers to a person, other than an Aboriginal person, who is non-Caucasian in race or non-white in colour.

#### ii) Dependent variable

The dependent variable (observed number of cases) was the number of laboratory-confirmed *S.* Enteritidis human infections reported in iPHIS from January 1, 2007 through December 31, 2009 from the three PHUs within the GTA, aggregated to the FSA-level. Using age-and-sex-based population estimates from the 2006 Census, the expected number of cases for each FSA was calculated using indirect standardisation [39].

### Modeling approach

A database containing the SES variables, dependent variable, and expected number of cases for each FSA was constructed in Microsoft Office Excel 2010, and subsequently imported into STATA Intercooled 10.1 statistical software (Stata Corporation, College Station, TX, US) for analysis. We initially analyzed the data using a Poisson regression model. To estimate SIRs of *S*. Enteritidis infections, the natural log-transformed expected number of cases was used as the offset to account for differences in FSA-level age-and-sex-based population that might affect FSA-level infection rates [40].

We assessed the relationship between the natural log of the SIR of *S.* Enteritidis infections and each of the continuous SES variables by a locally weighted regression method using lowess curves. If non-linearity was observed, we introduced a quadratic term. If the quadratic term was statistically significant (p ≤ 0.05), collinearity between the linear and quadratic terms was reduced by subtracting the mean value from each individual value of the linear term (centering), and then squaring each centered value [41]. If the non-linear relationship was not adequately modelled with a quadratic term, the variable was categorized into three equal groups - high, medium, and low - based on its distribution over all FSAs. The medium category was chosen as the reference category.

Univariable screening (α = 0.10) consisted of individually regressing each SES variable on the dependent variable. Pair-wise correlation coefficients using the Spearman’s rank test among all significant independent variables (p < 0.10) were examined to prevent the inclusion of collinear variables in the multivariable model. If two variables were highly correlated (rho > 0.70), the variable with the smallest p-value on univariable analysis was considered for the multivariable model.

All unconditionally significant variables identified on univariable screening, and a categorical variable signifying the three PHUs, were offered to a multivariable model, and a manual backward elimination process was performed. Variables with p > 0.05 were removed unless there was evidence of confounding (when removal of a non-intervening variable changed the coefficients of remaining variables by more than 25%) [41]. Once a main effects model was created, two-way interaction terms between significant SES variables were tested and retained in the final model if they were significant (p ≤ 0.05).

The fit of the Poisson model was assessed using the Pearson χ^2^ goodness-of-fit test [42]. The test was significant (χ^2^ = 178.84, P = 0.03) indicating that the model did not fit the data; therefore, the analysis was repeated using a negative binomial regression model.

The final negative binomial model was evaluated to assess model fit and to measure spatial autocorrelation among residuals. First, we assessed Ansombe residuals for each FSA to identify outliers, and then a normal quantile plot (quantiles of the residuals against the quantiles of the normal distribution) was used to assess model fit [43]. In addition, we visually assessed Ansombe residuals in a choropleth map using ArcGIS 10 to identify FSAs with over- or under-predicted values (> 3 or < − 3). Secondly, spatial autocorrelation of FSA-level residuals was evaluated by applying the global Moran’s I statistic [44]. A significant global Moran’s I (p ≤ 0.05) would indicate that FSA-level residuals are not randomly distributed across the study area and accounting for spatial clustering among FSAs would be required.

For each SES variable from the final model with a significant quadratic term, we plotted the variable (linear and quadratic terms) against the predicted log SIR of *S*. Enteritidis infections in order to describe the curvilinear relationship while controlling for the other variables in the model. For each SES variable from the final model, choropleth maps were created in ArcGIS 10 to visualize the FSA-level distribution of the data.

## Results

### Descriptive statistics

**Figure 2:**
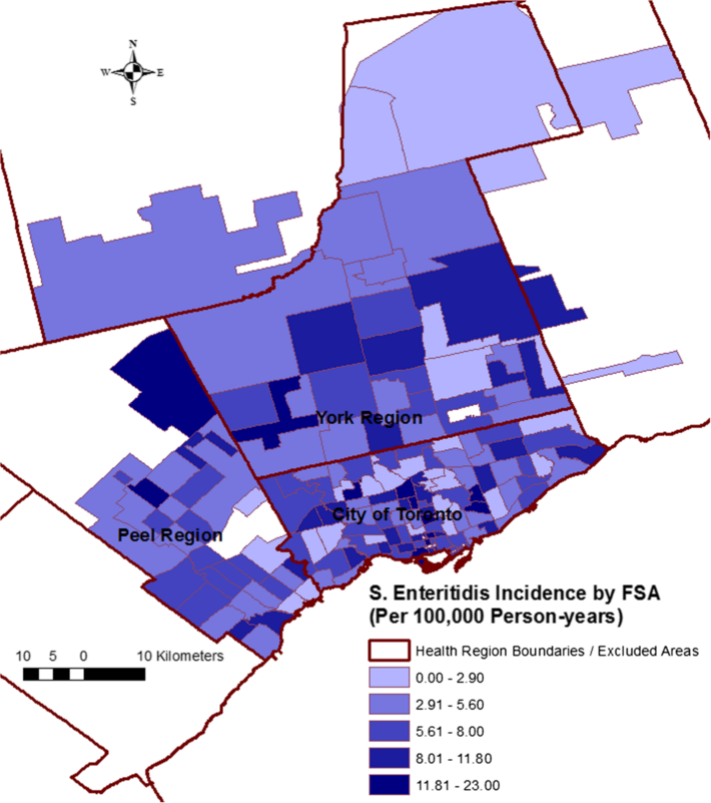
**Unadjusted *Salmonella *Enteritidis incidence per 100,000 person-years, by forward sortation area (FSA). **^a).^846 cases from 153 FSAs from three public health units (City of Toronto Health Unit, Peel Regional Health Unit, and York Regional Health Unit) within the Greater Toronto Area, Ontario, Canada were reported in the integrated Public Health Information System from January 1, 2007 through December 31, 2009 and included in the study.

No recurrent episodes of *S.* Enteritidis infections were identified. The total number of *S.* Enteritidis infections for the study period was 846; the count per FSA ranged from 0 to 18 (mean = 5.53), and the unadjusted incidence per 100,000 person-years per FSA over the study period ranged from 0 to 23 (mean = 6.45) (Figure 2).

**Figure 3:**
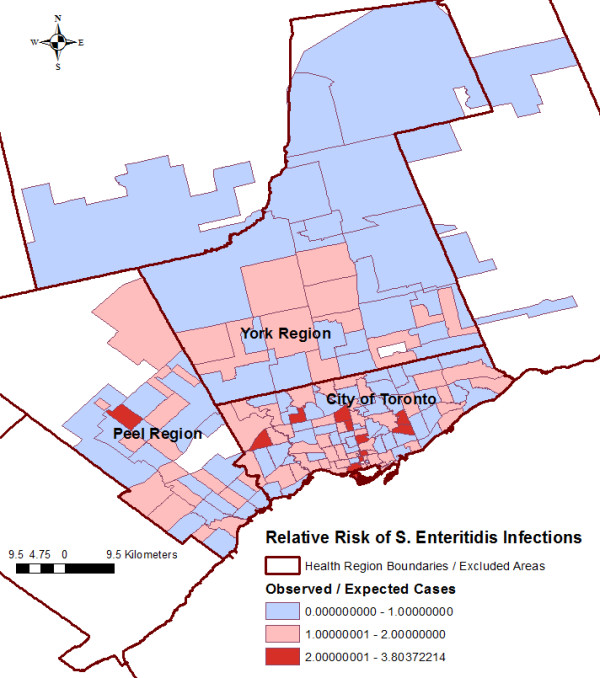
**Relative risk**^**a) **^**of *Salmonella *Enteritidis infections by forward sortation area. **^a)^ The ratio of the observed number of cases to the age-and-sex-adjusted expected number of cases. Values < 1 indicate lower incidence than expected, whereas values >1 indicate higher incidence than expected.

Figure 3 illustrates the FSA-level distribution of the relative risk (ratio of the observed number of cases to the age-and-sex-adjusted expected number of cases). Values < 1 indicate lower incidence than expected, whereas values > 1 indicate higher incidence than expected. There were 11 FSAs with a relative risk greater than 2.

### Spatial clustering

**Figure 4:**
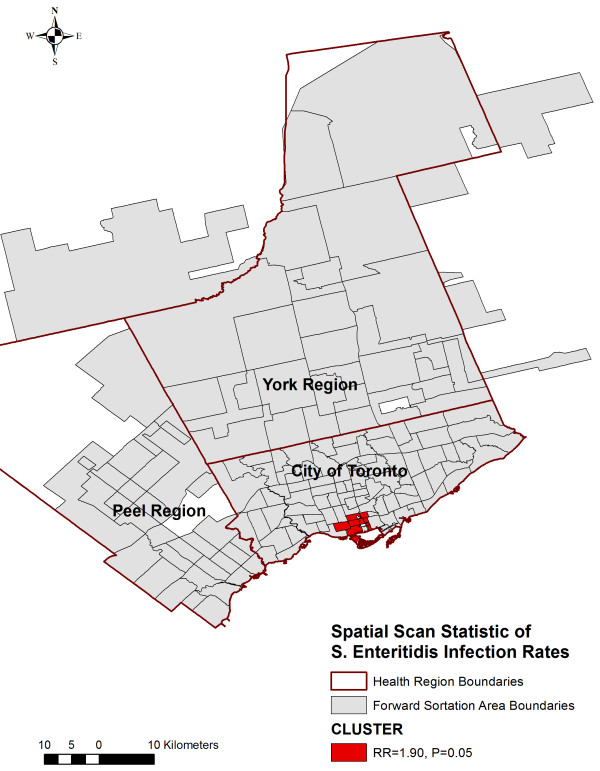
**Spatial cluster **^**a) **^**of high *Salmonella *Enteritidis infection rates. **^a^) Results of a spatial discrete Poisson model using the SatScanTM software. Study period: January 1, 2007 to December 31, 2009. A circular scanning window containing up to 50% of the population at risk was used. Age groups (0–9, 10–24, 25–34, 35–49, and 50+ years) and sex were included as covariates to account for confounding. Significant at p ≤ 0.05.

We identified a single cluster of higher than expected infection rates located in the south-central area (downtown) of the City of Toronto Health Unit, which included nine neighbouring FSAs [Relative Risk = 1.90, P = 0.050] (Table 1; Figure 4).

**Table 1:** **Spatial cluster of high *Salmonella *** **Enteritidis infection rates in the Greater Toronto Area, Ontario, Canada **^**a)**^

**Location Ids (Forward Sortation Areas)**	**Observed cases**	**Expected cases**	**Observed / Expected**	**Relative risk**	**Log likelihood ratio**	**P-value**
M5T, M5G, M5S, M5B, M6J, M5V, M5C, M4Y, M5E	40	21.56	1.85	1.90	6.49	0.050

^a)^ Results of a spatial discrete Poisson model using the SatScanTM software. A circular scanning window containing up to 50% of the population at risk was used. Age groups (0–9, 10–24, 25–34, 35–49, and 50+ years) and sex were used as covariates to account for confounding. Significant at p ≤ 0.05.

### Negative binomial regression

Variables significantly associated with the SIR of *S.* Enteritidis infections at the FSA-level on univariable screening included average number of children at home per census family, average number of persons per census family, average number of rooms per home, immigrant population proportion, average median family income, and visible minority population proportion (Table 2). The average number of children at home per census family and the average number of persons per census family were highly correlated (rho = 0.94, P < 0.01); average number of children at home was kept for the multivariable model because of its smaller p-value on univariable analysis and because children were identified as having the highest incidence rate of *S.* Enteritidis infections in Ontario during the study period [29].

**Table 2:** Results of univariable negative binomial regression models (n = 846 cases from 153 FSAs)

**Variable **^**a)**^	**Type**		**IRR **^**c) **^**(95% CI)**	**P - value**
**Average number of children at home per census family**	Linear (X)		1.03 (0.71, 1.50)	0.881
	Quadratic (X^^2^) ^b)^		4.45 (1.81, 10.92)	0.001
**Average number of persons per census family**	Linear (X)		0.89 (0.64, 1.25)	0.515
	Quadratic (X^^2^)		4.47 (1.82, 10.98)	0.001
**Average number of rooms per home**	Linear (X)		0.97 (0.91, 1.04)	0.398
	Quadratic (X^^2^)		1.08 (1.03, 1.13)	0.002
**Immigrant population proportion**	Linear (X)		0.99 (0.99, 1.00)	0.284
	Quadratic (X^^2^)		0.99 (0.99, 0.99)	0.014
**Average median family income**	Categorical	Low (< CAD ^d)^ 65,000)	1.30 (1.08, 1.57)	0.005
		Medium (CAD 65,000 - 85,000)	Reference	-
		High (> CAD 85,000)	1.31 (1.08, 1.58)	0.006
**Proportion of university graduates of persons between 25 to 64 years of age**	Categorical	Low (10.4 - 28.6)	0.93 (0.77, 1.12)	0.446
		Medium (28.7 – 40.0)	Reference	-
		High (40.4 – 72.5)	0.95 (0.78, 1.16)	0.640
**Unemployment rate of persons 15 years of age or older**	Categorical	Low (0 - 6.0)	1.06 (0.87, 1.29)	0.544
		Medium (6.1 - 7.5)	Reference	-
		High (7.6 - 11.0)	1.08 (0.89, 1.32)	0.423
**Visible minority population proportion**	Categorical	Low (2.1 - 29.4)	0.78 (0.64, 0.94)	0.009
		Medium (29.5 - 51.8)	Reference	-
		High (51.9 - 93.4)	0.77 (0.64, 0.92)	0.004

^a)^ Dependent variable: Number of *Salmonella* Enteritidis infections by forward sortation area (FSA). Offset: natural log-transformed FSA-based expected number of cases. ^b)^ Each linear (continuous) variable was centered by substracting the mean value from each value. A quadratic term for the centered linear variable was introduced and kept in the model if it was significant. If the linear and quadratic terms were not statistically significant, the variable was categorized into three equal groups (low, medium, and high). ^c)^ IRR: incidence rate ratio; CI: confidence interval. ^d)^ CAD: Canadian Dollar. Significant at p ≤ 0.05.

**Figure 5:**
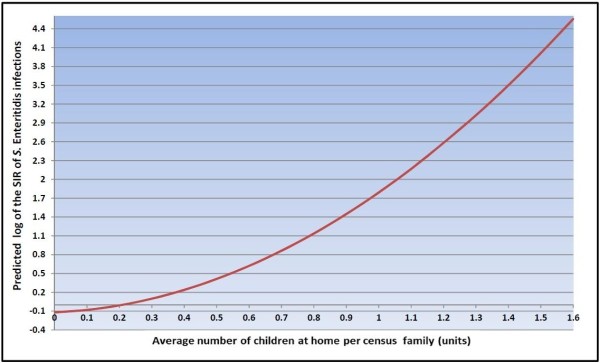
**Association between average number of children at home per census family and *Salmonella *Enteritidis infections **^**a)**^**. **^a)^ The graph displays the effect of a 0.1 unit change in the average number of children at home per census family on the predicted log of the standardized incidence rate (SIR) of *Salmonella* Enteritidis (*S*. Enteritidis) infections, keeping all other variables in the final negative binomial regression model at their referent level. The final model included the linear and quadratic terms of average number of children at home per census family, average median family income, and visible minority population proportion. Dependent variable: Number of *S*. Enteritidis infections by forward sortation area (FSA). Offset: natural log-transformed FSA-based expected number of cases.

**Figure 6:**
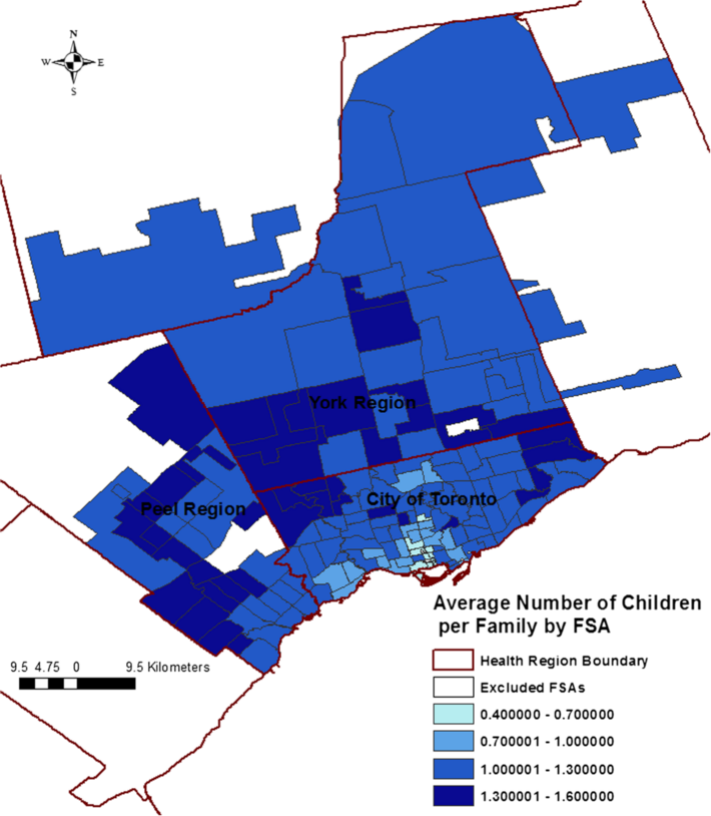
Distribution of the average number of children at home per census family by forward sortation area (FSA).

**Figure 7:**
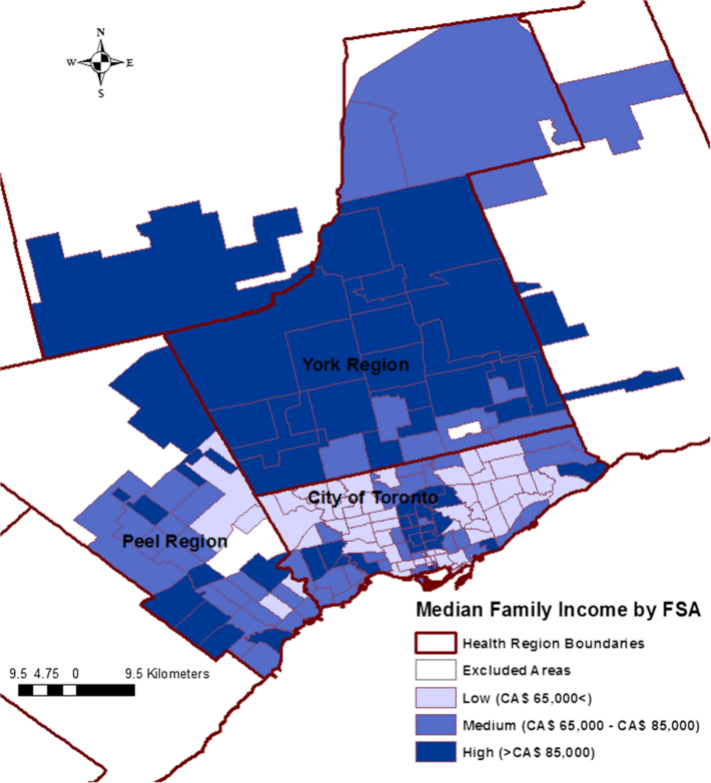
Distribution of the average median family income by forward sortation area (FSA).

**Figure 8:**
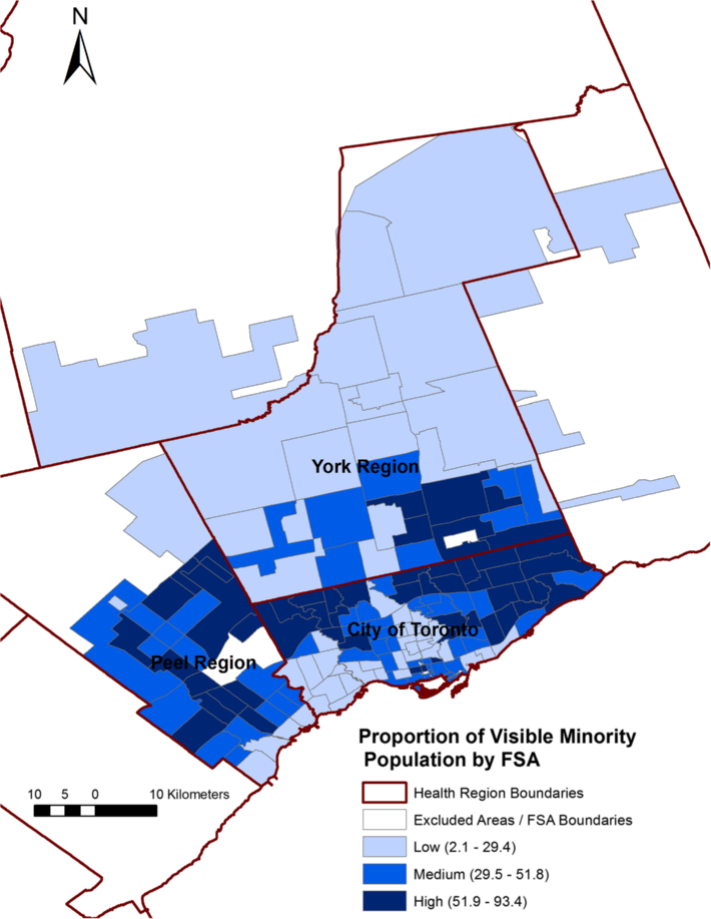
Distribution of the visible minority population proportion by forward sortation area (FSA).

Variables significantly associated with the SIR of *S.* Enteritidis infections at the FSA-level in the final multivariable model included average number of children at home per census family, average median family income, and visible minority population proportion (Table 3). No significant interaction terms were identified. Public health unit was not significant on the likelihood ratio (LR) test (LR χ^2^ = 2.77, P = 0.250); therefore, we did not keep this variable in our final model. Figure 5 shows a plot of the average number of children at home per census family against the predicted log SIR of *S*. Enteritidis infections, holding all other variables from the final model at their referent level. The positive curvilinear convex shape of the line indicates an exponential increase in the SIR of *S*. Enteritidis infections as the FSA-level average number of children at home per census family increases. Forward sortation areas with low average median family income [Incidence Rate Ratio (IRR): 1.34, P = 0.002] and high average median family income [IRR: 1.24, P = 0.020] had higher SIRs of *S.* Enteritidis infections than FSAs with medium average median family income. Forward sortation areas with high visible minority population proportion [IRR: 0.76, P = 0.002] had a lower SIR of *S.* Enteritidis infections than FSAs with medium visible minority population proportion. Figures 6, 7, and 8 display the FSA-level distribution of the average number of children at home per census family, average median family income, and visible minority population proportion, respectively. Visual assessment of the maps showed that there were clusters of high values for the FSA-level average number of children at home per census family in the north-western and eastern parts of the City of Toronto Health Unit, central and southern parts of the York Regional Health Unit, and most of the Peel Regional Health Unit except the southern part. Low average median family income FSAs clustered in the north-western, south-central, and eastern parts of the City of Toronto Health Unit, and in the mid-eastern part of the Peel Regional Health Unit. High average median family income FSAs clustered in the central part of the City of Toronto Health Unit, and in most of York Regional Health Unit except the southern part. Forward sortation areas with medium visible minority population proportion clustered in central parts of the City of Toronto Health Unit, and western parts of Peel Regional Health Unit.

**Table 3:** Results of the final multivariable negative binomial regression model (n = 846 cases from 153 FSAs)

**Variable **^**a)**^	**Type**		**Estimate (95% CI)**	**IRR **^**c) **^**(95% CI)**	**P-value**
**Average number of children at home per census family**	Linear (X) ^b)^		0.22 (−0.20, 0.64)	1.24 (0.82, 1.89)	0.313
	Quadratic (X^^2^)		1.69 (0.84, 2.53)	5.40 (2.32, 12.54)	<0.001
**Average median family income**	Categorical	Low (< CAD ^d)^ 65,000)	0.29 (0.11, 0.47)	1.34 (1.12, 1.61)	0.002
		Medium (CAD 65,000 - 85,000)	Reference	-	-
		High (> CAD 85,000)	0.22 (0.03, 0.40)	1.24 (1.03, 1.49)	0.020
**Visible minority population proportion**	Categorical	Low (2.1 - 29.4)	−0.16 (−0.35, 0.036)	0.85 (0.70, 1.04)	0.110
		Medium (29.5 - 51.8)	Reference	-	-
		High (51.9 - 93.4)	−0.27 (−0.44, -0.10)	0.76 (0.64, 0.91)	0.002
**Intercept**	-	-	−0.12 (−0.29, 0.05)	-	0.178

^a)^ Dependent variable: Number of *Salmonella* Enteritidis infections by forward sortation area (FSA). Offset: natural log-transformed FSA-based expected number of cases. ^b)^ Each linear (continuous) variable was centered by substracting the mean value from each value. A quadratic term for the centered linear variable was introduced and kept in the model if it was significant. If the linear and quadratic terms were not statistically significant, the variable was categorized into three equal groups (low, medium, and high). ^c)^ IRR: incidence rate ratio; CI: confidence interval. ^d)^ CAD: Canadian Dollar. Significant at P ≤ 0.05.

**Figure 9:**
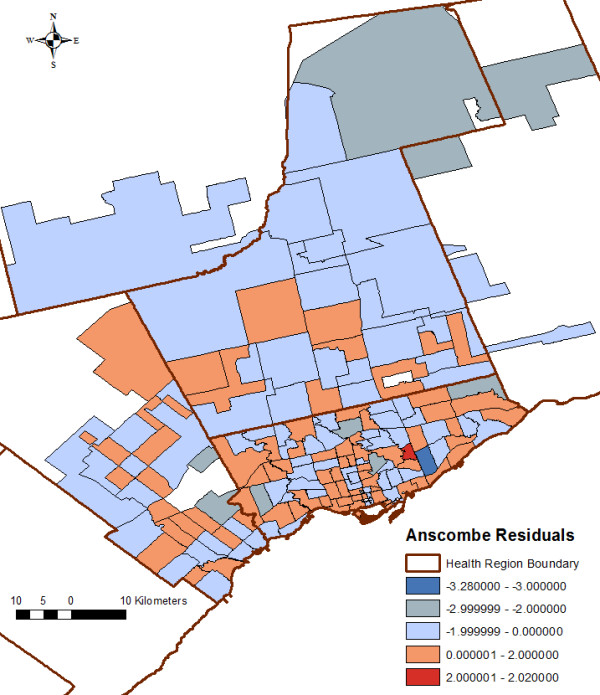
**Distribution of forward sortation area (FSA)-level Anscombe residuals from the multivariable negative binomial regression model **^**a)**^**. **^a^) Positive values signify under-prediction whereas negative values signify over-prediction. Dependent variable: Number of *Salmonella* Enteritidis infections aggregated to the Forward Sortation Area (FSA)-level. Offset: natural log-transformed FSA-based expected number of cases. Independent categorical variables: FSA-level average median family income and visible minority population proportion. Independent continuous variable: FSA-level average number of children at home per census family linear and quadratic terms.

Visually inspecting a normal quantile residual plot, we concluded that the FSA-level Anscombe residuals from the final regression model were normally distributed, indicating the model fit the data. Figure 9 illustrates the spatial distribution of the residuals; visually, no clustering of over- or under-predicted values was evident. The global Moran’s I statistic of the Anscombe residuals was not significant [I = −0.007, P = 0.98] indicating that no significant spatial autocorrelation of the residuals was present after accounting for the SES variables in the model. One outlier was identified; however, we kept it in the model because re-running the model without that observation did not modify any of the variable coefficients.

## Discussion

Our study used a spatial scan statistic to identify high rate clusters of *S.* Enteritidis infections in three PHUs within the GTA in Ontario. Multivariable negative binomial regression was employed to identify associations between *S.* Enteritidis infections and SES variables to better understand the underlying SES risk factors that might contribute to the spatial clustering of *S.* Enteritidis infections. Our unit of analysis was FSAs from these PHUs, where the *S.* Enteritidis case data acquired from the Ontario Ministry of Health and Long-Term Care (MOHLTC) integrated Public Health Information System (iPHIS) database, and the SES information obtained from the 2006 Census of Canada, were aggregated, merged, and analyzed. We combined traditional statistical methods with GIS technology (choropleth maps) for the statistically significant high rate cluster and the distribution of statistically significant SES indicators, which was a useful approach that assisted with visualizing FSA-level associations across the GTA.

We found that FSAs with high and FSAs with low average median family income had a higher incidence of *S.* Enteritidis infections compared to FSAs with medium average median family income (the reference category). An ecological study conducted in the US demonstrated higher salmonellosis rates in areas with high family income population compared to areas with low family income population [25]. The researchers hypothesized that residents from higher income areas had greater access to healthcare, health-seeking behaviour, and pet ownership, and had eating behaviours that increased resident’s salmonellosis risk. While there could be any number of underlying individual-level factors to explain our findings, one additional hypothesis for increased salmonellosis rates in high income neighbourhoods might be residents’ more frequent international travel, which has been shown to be an important risk factor for salmonellosis [9,18-20]. In Ontario, international travel, eating at a food establishment, and eating at home were the top three exposure settings for *S.* Enteritidis infections at the individual level during the study period (unpublished data). In contrast to our study, the US study had the lowest salmonellosis rates in low median family income areas. This discrepancy might be partly explained by the fact that all Canadian citizens, permanent residents, and landed immigrants have access to provincially-funded healthcare, which likely eliminates or reduces bias related to healthcare accessibility, compared to the US, where a large proportion of the population does not have access to funded healthcare [45]. Moreover, the US study combined all *Salmonella* serotypes. The pathogenicity and source attribution of *Salmonella* serotypes can differ [20,46,47], which might have led to non serotype-specific associations in the US study that are not representative of *S.* Enteritidis infections. Additionally, the higher rates of infection from low income areas in our study might be explained by poorer microbial quality of foods consumed [48], or by more frequent retail food safety violations [49,50] that could increase residents’ risk of foodborne diseases, including *S.* Enteritidis infections. Future research should consider public health inspection violations in different retail settings (e.g. stores, restaurants, food suppliers) to determine if there is an FSA-level association between *S.* Enteritidis infection rates and health inspection violations, and whether the association depends on average median family income or other FSA-level SES factors.

We found a positive curvilinear association between the FSA-level average number of children at home per census family and the SIR of *S.* Enteritidis infections. This finding could be explained by children’s higher susceptibility to salmonellosis, which has been demonstrated by individual-level studies [29,51,52]. Risk factors associated with *S.* Enteritidis infections in children include international travel [53,54], riding in shopping carts and exposure to raw meat and poultry products [55], person-to-person transmission in daycare centres and in private homes [56], and contact with reptiles [54,57,58] and cats [57]. In addition, an ecological study conducted in the US identified a positive association between salmonellosis and counties with a high proportion of young children, which appears to be a consistent risk factor for all *Salmonella* serotypes [28]. This finding might be explained by the higher severity of salmonellosis in this age group, and consequently an increased likelihood of visiting a physician and getting tested and reported [28]. However, we acknowledge that the average number of children at home per census family and the average number of persons per census family could be substitutes for each other due to their high correlation, which might suggest that home density and eating habits of larger families might have a role in the positive association with the SIR of *S.* Enteritidis infections. Future individual-level research is needed in Ontario to elucidate our finding.

Forward sortation areas with high visible minority population proportion had a lower SIR of *S.* Enteritidis infections compared to FSAs with medium visible minority population proportion. This finding is in contrast with a previous US ecological study, in which researchers demonstrated increased salmonellosis rates in areas with higher black, Hispanic, or Latino populations compared to areas with a predominantly Caucasian population [28]. However, comparing non-Caucasian populations between Canada and the US should be done with caution, because in the GTA, a high proportion of the non-Caucasian population is South Asian or Chinese [59]. Of note, in our study no significant difference was found between the low and high visible minority population proportion FSAs, which warrants further individual-level studies to better understand the effect of ethnicity on the incidence of *S.* Enteritidis infections.

A single spatial cluster of higher than expected incidence rates of *S.* Enteritidis infections was identified in the south-central area (downtown) of the City of Toronto Health Unit that included nine neighbouring FSAs. The majority of these FSAs had SES characteristics that were positively associated with the SIR of *S.* Enteritidis infections in the regression model (low average median family income, medium visible minority population proportion), which is consistent with these SES indicators playing a significant role in the clustering of *S.* Enteritidis infections. However, many of the FSAs in this cluster had one SES characteristic that was inconsistent with the regression model (low average number of children at home per census family), which warrants further spatial epidemiological assessment. Applying GIS technology to visualize the FSA-level spatial cluster of high incidence rates of *S.* Enteritidis infections and the FSA-level distribution of significant SES variables was an useful technique to highlight areas where prevention and control programs should be targeted, and where future studies should be conducted to understand the underlying individual-level risk factors.

First- and second-order spatial effects must be considered when analyzing spatial epidemiological data [39]. First-order effects in our analysis could be defined as variation in the mean value of the *S.* Enteritidis infection rate in the study area (i.e. a global or large-scale trend). We addressed these effects by using a multivariable negative binomial regression model. Second-order effects in our study could be defined as spatial dependence in the *S*. Enteritidis infection rates (i.e. local or small-scale effects). It could be expected that neighbouring FSAs have more common infection rates and SES indicator features than distant FSAs. We assessed the presence of second-order effects by evaluating the spatial autocorrelation of FSA-level Anscombe residuals using the global Morans’s I statistic. The statistic was not significant indicating that the SES variables explained the variation in *S.* Enteritidis infection rates across the study area well and no second-order effects remained once these fixed effects were accounted for in our model.

Before generalizing our findings, a few limitations need to be considered. Passive laboratory-based surveillance programs underestimate the true burden of enteric infections. In Canada, it was estimated that for every reported salmonellosis case there were 26.1 cases in the general population that remained unreported [60]. There might also be geographic variations in under-reporting of *S.* Enteritidis infections due to differences in utilization of health care providers or the sensitivity of testing methods used at different laboratories [61,62]. However, in our study, these factors might not be as influential because our study area enclosed three neighbouring PHUs with similar healthcare providers.

A second limitation of our study is that we were unable to adjust our estimates for the seasonal variation in the incidence rate of *S.* Entertidis infections [29]. To account for month and year in our analysis we would need to have area-level age-and-sex-based monthly and yearly population estimates, and monthly and yearly socioeconomic status estimates. Socioeconomic data are collected every five years in Canada and the data are not subdivided into smaller time units (e.g. month or year).

Another possible limitation is that ecological studies only consider variation between groups and not within groups [63], making them unsuitable to relate population level risk factors to the individual level. However, ecological studies can be considered as a cost-effective and useful alternative to individual-level studies, where regional differences in SES risk factors can be evaluated and identified, which can be used by public health authorities to further assess these risk factors and target prevention and control programs [25,28]. It is also recognized that analyzing associations among *S.* Enteritidis infections and SES variables at higher geographic levels (e.g. health unit, province) might have given different results [64] due to the Modifiable Areal Unit problem. However, by analyzing the data at a lower geographic level (in our case, the FSA-level), we have likely decreased the ecological bias (by reducing the variation of SES factors within each region), and obtained more conservative estimates of our regression coefficients [65].

Our main objective was to identify area-level associations between *S.* Enteritidis infection rates and SES indicators. Several variables that we investigated were based on census families (average number of children per census family, average number of persons per census family, average median family income); consequently, for these three SES indicators, single person homes were not investigated in our study, which could limit the external validity of our estimates. However, within the study area, the proportion of single person households was considerably lower than census family households [33].

Finally, we analyzed *S.* Enteritidis case data from 2007 to 2009; however, the only available socioeconomic data were from the 2006 Census of Canada. Population changes over the study period could have introduced bias into our study. However, it is likely that SES indicators and population sizes did not change drastically over this relatively short time period. We excluded FSAs with a population of less than 500 residents; however, these areas accounted for less than one percent of the total population, and therefore it is unlikely that these exclusions affected our results.

## Conclusions

Our study demonstrated the usefulness of combining GIS technology and conventional and spatial statistical methods to identify high rate clusters and to analyze area-level associations between *S.* Enteritidis infection rates and SES variables in three PHUs within the GTA by using an ecological study approach. We found a higher incidence rate of *S.* Enteritidis infections in FSAs with low and high average median family income compared to FSAs with medium average median family income that might be explained by the poorer quality of food consumed in the low income areas, in differences in food preparation and consumption practices among various areas, and in high income area residents’ more frequent international travel that increases their risk of salmonellosis. A positive curvilinear relationship was observed between the FSA-level SIR of *S.* Enteritidis infections and average number of children at home per census family that might be attributable to children’s greater susceptibility to enteric infections. We did not detect any significant spatial dependency of residuals indicating that our fixed effects model explained the spatial dependency well.

The high rate cluster of *S.* Enteritidis infections detected by the scan statistic contained mainly FSAs in which the majority of the SES variables identified in the regression model were positively associated with *S.* Enteritidis infections, suggesting that these SES indicators (low average median family income, medium visible minority population proportion) significantly contributed to the spatial clustering of *S.* Enteritidis infections.

These findings will aid public health policy makers and practitioners to further evaluate individual-level SES indicators. Areas with low and high average median family income, medium proportion of visible minority population, and high average number of children at home per census family should be targeted when designing disease control and prevention programs within these PHUs. Further studies are needed in areas with high *S.* Enteritidis infection rates to identify sources of environmental contamination of the local food supply, to assess food safety practices at local food markets, retail stores, and restaurants, to identify novel individual-level risk factors, and to detect spatio-temporal trends of *S.* Enteritidis infections.

## Abbreviations

CI: Confidence interval; FSA: Forward sortation area; GIS: Geographic Information System; GTA: Greater Toronto Area; iPHIS: integrated Public Health Information System; IRR: Incidence rate ratio; LR: Likelihood ratio; MOHLTC: Ontario Ministry of Health and Long-Term Care; PHU: Public Health Unit; S. Enteritidis: *Salmonella enterica subspecies enterica* serotype Enteritidis; SES: Socioeconomic status; SIR: Standardized incidence rate; US: United States of America.

## Competing interests

The authors declare that they have no competing interests.

## Authors’ contributions

CV developed the study design, analyzed the data, interpreted results, wrote the first draft of the manuscript, responded to editorial comments, and prepared the final manuscript for submission. MTG and DLP were consulted on data analysis, study design, interpretation of results, and reviewed and commented on manuscript drafts. SAM, FP, and JMS provided advice on the data analysis, interpretation of results, and reviewed and commented on manuscript drafts. All authors read and approved the final manuscript.

## Pre-publication history

The pre-publication history for this paper can be accessed here:

http://www.biomedcentral.com/1471-2458/13/1078/prepub
